# Supplementary material for: Clothianidin seed-treatment has no detectable negative impact on honeybee colonies and their pathogens
Source: Nat Commun. 2019 Feb 11;10:692. doi: 10.1038/s41467-019-08523-4 (PMC6370849; doi:10.1038/s41467-019-08523-4)
Supplement: Supplementary file 1 — Supplementary Information [file 41467_2019_8523_MOESM1_ESM.pdf]

Supplementary Information for

Clothianidin seed-treatment has no detectable negative impact on honeybee colonies and their pathogens

Osterman *et al*.

Correspondence to: jul.osterman@gmail.com

This PDF file includes:

Supplementary Table 1 - 13

Supplementary Figure 1 – 3

Supplementary Methods

Supplementary References

Supplementary Tables

Supplementary Table 1 | Number of positive and negative samples per year and target organism

Target Organism*	Year**	Number of samples‡	Number of positive samples	Number of negative samples	Statistical analysis prevalence§	Statistical analysis abundance§§
Gilliamella apicola	2013	154	154	0	No	Yes
	2014	74	74	0	No	Yes
Snodgrassella alvi	2013	154	153	1	No	Yes
	2014	74	74	0	No	Yes
Acute bee paralysis virus (ABPV)	2013	154	8	146	No	No
	2014	74	51	23	Yes	Yes
Aphid lethal paralysis virus (ALPV)	2013	154	24	130	Yes	Yes
	2014	74	23	51	Yes	Yes
Black queen cell virus (BQCV)	2013	154	154	0	No	Yes
	2014	74	72	2	No	Yes
Big Sioux River virus (BSRV)	2013	154	7	147	No	No
	2014	74	1	73	No	No
Chronic bee paralysis virus (CBPV)	2013	154	1	153	No	No
	2014	74	32	42	Yes	Yes
Deformed wing virus type-A (DWV-A)	2013	154	24	130	Yes	Yes
	2014	74	40	34	Yes	Yes
Deformed wing virus type-B (DWV-B)	2013	154	72	82	Yes	Yes
	2014	74	24	50	Yes	Yes
Israeli acute paralysis virus (IAPV)	2013	154	0	154	No	No
	2014	74	14	60	Yes	Yes
Kashmir bee virus (KBV)	2013	154	0	154	No	No
	2014	74	27	47	Yes	Yes
Lake Sinai virus type-1 (LSV-1)	2013	154	153	1	No	Yes
	2014	74	67	7	No	Yes
Lake Sinai virus type-2 (LSV-2)	2013	154	2	152	No	No
	2014	74	5	69	No	No
Slow bee paralysis virus (SBPV)	2013	154	1	153	No	No
	2014	74	72	2	No	Yes
Sacbrood virus (SBV)	2013	154	148	6	No	Yes
	2014	74	68	6	No	Yes
Nosema apis	2013	154	42	113	Yes	Yes
	2014	74	7	67	No	No
Nosema ceranae	2013	154	82	72	Yes	Yes
	2014	74	48	26	Yes	Yes
Varroa destructor	2013	154	50	104	Yes	Yes
	2014	74	70	4	No	Yes

* Species names are spelled in italics.
** Target organism was analysed in a full model (2013 and 2014) of sample size was >9 in both years for prevalence.
‡ Number of samples in 2013 (38 samples were excluded from the 192 (96 before and 96 after oilseed rape bloom) samples due to swarming (2 x 18) or loss of queen (2 x 1)) and in 2014 (6 samples were excluded from the 80 samples (2 x 40) due to swarming (2 x 3)).
§ If the data consisted of a sample size < 10 (positive or negative sample size) it was not statistically analysed for prevalence.
§§ If the data consisted of <10 positive samples it was not statistically analysed for abundance.

Supplementary Table 2 | Residues of neonicotinoids in honeybees, pollen and nectar*

	**Control (2013: n = 8**; 2014: n = 4)**		**Clothianidin treated (2013: n = 8; 2014: n = 6)**			
	**Detected in n samples**	**Highest concentration**	**Detected in n samples**	**Highest concentration**	**LOD †**	**LOQ †**
**2013**						
**Honeybee pollen (ng g^{-1})**						
Acetamiprid	1	0.34	0		0.080	0.24
Clothianidin	0		8	23	0.50	1.5
Imidacloprid	1	0.23††	0		0.30	0.90
Thiacloprid	3	1.4§	4	0.29	0.070	0.21
Thiametoxam	0		0		0.10	0.30
**Honeybee nectar (ng ml^{-1})**						
Acetamiprid	0		0		0.033	0.10
Clothianidin	2	0.61	8	16	0.17	0.50
Imidacloprid	3	0.35	0		0.17	0.50
Thiacloprid	2	0.35§	2	0.044	0.033	0.10
Thiametoxam	1	0.19	0		0.17	0.50
**Honeybee tissue (ng g^{-1})**						
Acetamiprid	1	0.012††	0		0.020	0.060
Clothianidin	2	0.89	8	4.9	0.080	0.25
Imidacloprid	0		0		0.040	0.12
Thiacloprid	2	0.058§	2	1.1	0.030	0.090
Thiametoxam	1	0.19	0		0.070	0.20
**2014**						
**Honeybee pollen (ng g^{-1})**						
Acetamiprid	1	0.18	0		0.1	0.3
Clothianidin	0		6	16	0.25	0.75
Imidacloprid	0		1	0.39	0.2	0.6
Thiacloprid	3	1.3	2	358§§	0.025	0.075
Thiametoxam	0		0		0.06	0.2
**Honeybee nectar (ng ml^{-1})**						
Acetamiprid	0		1	0.68	0.033	0.1
Clothianidin	0		6	9.8	0.17	0.5
Imidacloprid	0		1	1.3	0.17	0.5
Thiacloprid	1	0.06	2	15§§	0.033	0.1
Thiametoxam	0		1	0.2	0.17	0.5
**Honeybee tissue (ng g^{-1})**						
Acetamiprid	0		1	0.026	0.02	0.06
Clothianidin	0		6	1.5	0.08	0.25
Imidacloprid	1	0.57	0		0.04	0.12
Thiacloprid	1	0.043	3	6.9§§	0.03	0.09
Thiametoxam	0		0		0.07	0.2

* Residues of neonicotinoids in honeybee- collected pollen and nectar and honeybee tissue from control fields and fields sown with clothianidin treated seeds in 2013 and 2014.
** n = 6 for pollen collected by honeybees at control fields, because no such bees with pollen could be found at two fields.
† LOD, limit of detection; LOQ, limit of quantification.
†† Sample weight of 0.091 g explains reported value slightly below the estimated limit of detection, based on a 0.056 g sample weight.
§ One oilseed rape field sprayed with Biscaya (12 June 2013), where thiacloprid is the active ingredient (Supplementary Table 8).
§§ One oilseed rape field sprayed with Biscaya (29 June 2014), where thiacloprid is the active ingredient (Supplementary Table 8).

Supplementary Table 3 | Honeybee colony mortality and re-queening for 2013 and 2014

		2013*				† 2013 - 2014				2014*			
		**Clothianidin Treated**		**Control**		**Clothianidin Treated**		**Control**		**ClothianidinTreated**		**Control**	
		**No.**	**%**	**No.**	**%**	**No.**	**%****	**No.**	**%****	**No.**	**%**	**No.**	**%**
**MORTALITY**	**Autumn decision‡**					**9**	**19**	**7**	**15**				
	re-queened					9	60	7	58				
	1-yr queen‡‡					0	0	0	0				
	2-yr queen‡‡					0	0	0	0				
	**Winter loss§**					**5**	**13**	**6**	**15**				
	re-queened					3	50	3	60				
	1-yr queen‡‡					2	7	3	9				
	2-yr queen‡‡					0	0	0	0				
	**Total loss**					**14**	**30**	**13**	**27**				
	swarmed/superseded					12	80	10	83				
	original queen					2	6	3	8				
**REQUEENING§§**	**Queen-cells**	**16**	**34**	**16**	**33**	**12**	**75**	**10**	**63**	**3**	**13**	**2**	**13**
	1-yr queen‡‡	13	32	13	31	9	69	8	62	n.a.#	n.a.#	n.a.#	n.a.#
	2-yr queen‡‡	3	50	3	50	3	100	2	67	3	13	2	13
	**Swarmed**	**10**	**21**	**8**	**17**	**8**	**80**	**7**	**88**	**2**	**8**	**1**	**6**
	1-yr queen‡‡	8	20	8	19	6	75	7	88	n.a.#	n.a.#	n.a.#	n.a.#
	2-yr queen‡‡	2	33	0	0	2	100	0	n.a.	2	8	1	6
	**Superseded**	**5**	**11**	**4**	**8**	**4**	**80**	**3**	**75**	**0**	**0**	**0**	**0**
	1-yr queen‡‡	4	10	1	2	3	75	1	100	n.a.#	n.a.#	n.a.#	n.a.#
	2-yr queen‡‡	1	17	3	50	1	100	2	67	0	0	0	0

* Sample size: n = 8 fields per treatment in 2013, with 6 colonies per field; n = 4 control and n = clothianidin-treated fields in 2014, with 4 colonies per field.

** Mortality percentages are calculated with respect to the total number of available colonies at the point of time in the season for the main categories (**bold**), and with respect to the number of available colonies for each queen age-group for the sub-categories.

‡ Autumn decision (04 September 2013) refers to the number/proportion of 2013 experimental colonies deemed too weak to survive winter by the assessing beekeeper.

‡‡ 1-yr queen, one-year old queen; 2-yr queen, two-year old queen.

§ Winter loss (between 05 September 2013 and April 2014) refers to number/proportion of colonies lost out of those deemed strong enough to overwinter by the assessing beekeeper.

§§ For the 2013 and 2014 re-queening events, the proportions for the main category (**bold**) were calculated as a function of the total available treated or untreated colonies, whereas for the sub-categories it was calculated with respect to the number of available colonies in the specific queen-age subcategory. The 2013-2014 mortality data for this section refers exclusively to those colonies that had re-queened during 2013 that were no longer alive in April 2014, either through winter loss or autumn beekeeper assessment.

# Only colonies with one-year old queens throughout 2013 (i.e. two-year old queens during 2014) were used for the 2014 experiment.

Supplementary Table 4 | Pathogen and parasite prevalence in relation to fixed effects (seed treatment, bloom and year)*

Pathogen or Parasite**	Model type#	Effect measure	Estimate‡	DF	*χ2*	*P*‡
Acute bee paralysis virus	2014	Intercept	0.960			
		Seed-treatment	0.104	1	0.10	0.751
		Bloom	-0.375	1	1.85	0.174
		Seed-treatment x Bloom	-0.013	1	<0.01	0.964
Aphid lethal paralysis virus	Full model	Intercept	-3.935			
		Seed-treatment	2.737	1	0.97	0.324
		Bloom	3.396	1	21.36	**<0.001**
		Year	-2.076	1	7.11	**0.008**
		Seed-treatment x Bloom	-2.786	1	2.56	0.109
		Seed-treatment x Year	2.430	1	0.27	0.601
		Bloom x Year	2.727	1	1.24	0.265
		Seed-treatment x Bloom x Year	-2.676	1	2.83	0.093
Chronic bee paralysis virus	2014	Intercept	-0.328			
		Seed-treatment	0.013	1	<0.01	0.989
		Bloom	-0.457	1	2.18	0.140
		Seed-treatment x Bloom	0.351	1	1.76	0.185
Deformed wing virus type-A	Full model	Intercept	-1.135			
		Seed-treatment	-0.387	1	8.32	**0.004**
		Bloom	2.020	1	72.29	**<0.001**
		Year	1.666	1	55.66	**<0.001**
		Seed-treatment x Bloom	-0.188	1	0.56	0.455
		Seed-treatment x Year	0.477	1	1.98	0.159
		Bloom x Year	0.881	1	5.97	**0.015**
		Seed-treatment x Bloom x Year	0.338	1	1.16	0.280
Deformed wing virus type-B	Full model	Intercept	-0.549			
		Seed-treatment	0.010	1	<0.01	0.969
		Bloom	-0.425	1	30.12	**<0.001**
		Year	-0.307	1	4.86	**0.028**
		Seed-treatment x Bloom	-0.183	1	1.11	0.292
		Seed-treatment x Year	0.003	1	<0.01	0.980
		Bloom x Year	1.256	1	57.60	**<0.001**
		Seed-treatment x Bloom x Year	0.012	1	<0.01	0.947
Israeli acute paralysis virus	2014	Intercept	-1.616			
		Seed-treatment	-0.230	1	2.03	0.154
		Bloom	-0.626	1	3.35	0.067
		Seed-treatment x Bloom	0.626	1	3.37	0.066
Kashmir bee virus	2014	Intercept	-0.538			
		Seed-treatment	-0.258	1	1.06	0.304
		Bloom	-0.421	1	2.92	0.087
		Seed-treatment x Bloom	-0.0607	1	<0.01	0.977
Nosema apis	2013	Intercept	-11.959			
		Seed-treatment	-0.130	1	1.53	0.216
		Bloom	-12.080	1	72.61	**<0.001**
		Seed-treatment x Bloom	-0.154	1	<0.01	1.000
Nosema ceranae	Full model	Intercept	0.535			
		Seed-treatment	-0.587	1	13.82	**<0.001**
		Bloom	-1.414	1	90.22	**<0.001**
		Year	0.305	1	6.32	**0.012**
		Seed-treatment x Bloom	-0.129	1	0.28	0.597
		Seed-treatment x Year	0.289	1	1.56	0.211
		Bloom x Year	0.395	1	3.30	0.069
		Seed-treatment x Bloom x Year	-0.157	1	0.60	0.437
Varroa destructor	2013	Intercept	-0.878			
		Seed-treatment	-0.114	1	0.50	0.481
		Bloom	0.600	1	10.38	**0.001**
		Seed-treatment x Bloom	-0.165	1	0.75	0.388

* Pathogen and parasite prevalence in honeybee colonies in relation to clothianidin seed treatment, bloom (before and after oilseed rape bloom) and year (2013 or 2014).
** Species names are spelled in italics.
# Full model: n = 14, clothianidin-treated; n = 12, control fields, repeated measurements. 2013: n 0 8 per treatment. 2014: n = 6, clothianidin-treated; n = 4, control.
‡ Main effects/interactions were estimated using sum-to-zero contrasts and the deviation of the second level (After, Clothianidin, 2014) of each factor (Bloom: before/after, Seed-treatment: control/clothianidin, Year: 2013/2014) from to the grand mean (intercept) is presented. P values < 0.005 are highlighted in bold.

Supplementary Table 5 | mRNA transcript levels of various immune defense genes in relation to clothianidin seed seed-treatment and boom (before or after oilseed rape bloom) at apiary level in 2013.

**Gene expression***	**Effect measure**	**Estimates****	**Degrees of freedom**	***F***	***P*‡**
Amel\LRR	Intercept	6.649			
	Seed-treatment	0.013	1, 14	0.14	0.716
	Bloom	-0.024	1, 14	0.56	0.466
	Seed-treatment x Bloom	0.039	1, 14	1.48	0.243
Apidaecin	Intercept	9.802			
	Seed-treatment	-0.015	1, 14	0.10	0.758
	Bloom	0.120	1, 14	6.51	**0.023**
	Seed-treatment x Bloom	0.065	1, 14	1.93	0.187
cSP33	Intercept	8.133			
	Seed-treatment	-0.029	1, 14	0.26	0.617
	Bloom	0.092	1, 14	2.75	0.119
	Seed-treatment x Bloom	0.073	1, 14	1.71	0.211
Dorsal-1A	Intercept	7.406			
	Seed-treatment	-0.010	1, 14	0.05	0.823
	Bloom	0.013	1, 14	0.10	0.755
	Seed-treatment x Bloom	0.032	1, 14	0.56	0.468
Eater-like	Intercept	6.022			
	Seed-treatment	-0.039	1, 14	0.88	0.365
	Bloom	0.010	1, 14	0.06	0.813
	Seed-treatment x Bloom	0.013	1, 14	0.10	0.754
NimcC2	Intercept	8.078			
	Seed-treatment	-0.037	1, 14	0.46	0.510
	Bloom	0.073	1, 14	1.80	0.201
	Seed-treatment x Bloom	0.073	1, 14	1.80	0.200
PGRP-S2	Intercept	8.306			
	Seed-treatment	0.001	1, 14	0.00	0.987
	Bloom	-0.022	1, 14	0.23	0.640
	Seed-treatment x Bloom	0.019	1, 14	0.18	0.682
SPH51	Intercept	7.880			
	Seed-treatment	-0.008	1, 14	0.03	0.876
	Bloom	0.133	1, 14	8.02	**0.013**
	Seed-treatment x Bloom	0.005	1, 14	1.18	0.296

* Species names are spelled in italics.
** Main effects/interactions were estimated using sum-to-zero contrasts and the deviation of the second level (After, Clothianidin) of each factor (Bloom: before/after, Seed-treatment: control/clothianidin) from to the grand mean (intercept) is presented. N = 8 fields per treatment.
‡ P values < 0.05 are highlighted in bold.

Supplementary Table 6 | Average monthly temperature (°C), days with frost and precipitation (mm) at three weather stations (Helsingborg, Halmstad, Kristianstad) in the study region (see Fig. 1: Map) during April-June in 2013 and 2014[1]

		**Helsingborg**		**Halmstad**		**Kristianstad**	
		**2013**	**2014**	**2013**	**2014**	**2013**	**2014**
**Temperature**	April	6.0	9.1	5.2	9.0	5.4	8.1
	May	13.2	12.5	13.5	12.4	12.8	12.0
	June	15.4	15.4	15.2	15.3	15.7	15.0
**Days with frost**	April	11	1	13	5	13	6
	May	3	0	2	4	4	3
	June	0	1	0	1	0	0
**Precipitation**	April	25	42	29	50	24	23
	May	65	95	49	78	35	89
	June	76	51	111	64	73	70

Supplementary Table 7 | Field size and land use in the landscapes (radius = 2 km) surrounding the focal oilseed rape fields for 2014

	Untreated (n = 4)		Clothianidin treated (n = 6)		Test of difference between treatments		Correlation matrix (Pearson correlation coefficient, r)									
	mean ± s.e.m.	min-max	mean ± s.e.m.	min-max	F_{df}	P	Agricultural land	Annually tilled arable land	Semi-natural grassland	Length of permanent field borders	Maize cultivation	Spring sown oilseed rape	Winter sown oilseed rape	Mass-flowering crops*	Forest	Urban
Size of focal oilseed rape field (ha)	10.0 ± 3.4	5.0-20.0	8.7 ± 1.7	4.0-14.0	$0.15_{1, 8}$	0.71	0.610	0.740	-0.583	0.477	0.314	0.882	0.398	0.666	-0.562	-0.330
Agricultural land (%)	59.1 ± 11.2	27.2-76.9	52.8 ± 5.8	11.5-86.8	$0.29_{1, 8}$	0.73		0.896	-0.062	0.922	0.692	0.702	0.483	0.693	-0.992	-0.313
Annually tilled arable land (%)	26.5 ± 7.4	9.4-45.0	33.2 ± 12.5	2.7-65.7	$0.24_{1, 8}$	0.64			-0.441	0.684	0.589	0.856	0.553	0.851	-0.847	-0.376
Semi-natural grassland (%)	5.4 ± 1.4	1.9-8.5	2.9 ± 0.9	1.0-6.6	$2.35_{1, 8}$	0.16				0.225	0.159	-0.522	-0.084	-0.371	-0.021	0.265
Length of permanent field borders (km)	15.2 ± 2.0	1.0-18.5	12.3 ± 1.9	4.4-16.4	$1.04_{1, 8}$	0.34					0.625	0.500	0.269	0.489	-0.950	-0.244
Maize cultivation (%)	2.0 ± 0.8	0.8-4.1	1.5 ± 0.6	0-3.8	$0.24_{1, 8}$	0.64						0.539	0.544	0.512	-0.730	0.316
Spring sown oilseed rape (%) – including the focal field	0.9 ± 0.3	0.4-1.9	0.9 ± 0.3	0.2-2.1	$0.01_{1, 8}$	0.92							0.678	0.782	-0.648	-0.376
Winter sown oilseed rape (%)	1.2 ± 0.2	0.6-1.7	0.9 ± 0.8	0-4.8	$0.09_{1, 8}$	0.78								0.659	-0.426	-0.264
Mass-floweing crops* (%)	6.5 ± 0.5	5.4-7.8	6.2 ± 2.2	0.2-13.6	$0.01_{1, 8}$	0.93									-0.641	-0.234
Forest (%)	22.5 ± 11.9	4.4-56.2	31.6 ± 11.4	2.8-69.0	$0.28_{1, 8}$	0.61										0.221
Urban (%)	2.0 ± 1.4	0-5.9	1.0 ± 0.8	0-4.9	$0.45_{1, 8}$	0.52										

*Mass-flowering crops include potato (35%), oilseed rape (31%), pea (22%), fruit and berry cultivation (85%), bean (4%), herbs and seeds (<1%).

Supplementary Table 8 | Insecticide spray treatments in the oilseed rape fields during the 2013 and 2014 growing seasons

		Spray treatment 1			Spray treatment 2			Spray treatment 3				
**Pair**	**Seed treatment ***	**Date**	**Product**	**Dose**	**Date**	**Product**	**Dose**	**Date**	**Product**	**Dose**	**Swarm /Supers §**	**Winter loss‡**
		**2013**										
P01	untr	04 June	Mavrik	0.25 l ha^{-1}							3	3
P01	treat	06 June	Plenum	150 g ha^{-1}	15 June	Steward	85 g ha^{-1}				3	3
P02	untr	31 May	Plenum	160 g ha^{-1}	10 June	Mavrik	0.20 l ha^{-1}				0	1
P02	treat	04 June	Plenum	150 g ha^{-1}	10 June	Steward	85 g ha^{-1}				0	0
P03	untr	No treatment									3	2
P03	treat	12 June	Avaunt	170 g ha^{-1}							0	0
P04	untr	16 June	Avaunt	160 g ha^{-1}							2	3
P04	treat	07 June	Plenum	150 g ha^{-1}							0	0
P05	untr	12 June	Plenum	150 g ha^{-1}							0	0
P05	treat	30 May	Plenum	150 g ha^{-1}							4	3
P06	untr	12 June	Biscaya	0.30 l ha^{-1}	19 June	Mavrik	0.25 l ha^{-1}				1	2
P06	treat	07 June	Avaunt	170 g ha^{-1}							5	5
P07	untr	04 June	Avaunt	170 g ha^{-1}	08 June	Plenum	150 g ha^{-1}				3	2
P07	treat	31 May	Plenum	150 g ha^{-1}							3	2
P08	untr	30 May	Avaunt	170 g ha^{-1}							0	0
P08	treat	04 June	Plenum	150 g ha^{-1}	14 June	Avaunt	120 g ha^{-1}				0	1
		**2014**										
P01	treat	15 June	Mavrik	0.25 l ha^{-1}							1	x
P01	untr	31 May	Mavrik	0.30 l ha^{-1}	6 June	Avaunt	0,20 l ha^{-1}				1	x
P02	treat	29 June	Biscaya	0.30 l ha^{-1}							0	x
P04	untr	28 May	Plenum	150 g ha^{-1}	9 June	Mavrik	0.20 l ha^{-1}				0	x
P04	treat	14 June	Avaunt	170 g ha^{-1}							0	x
P05	untr	24 May	Mavrik	0.25 l ha^{-1}	2 June	Plenum	150 g ha^{-1}	10 June	Mavrik	0.25 l ha^{-1}	0	x
P05	treat	5 June	Plenum	150 g ha^{-1}	8 June	Mavrik	0.20 l ha^{-1}				0	x
P06	treat	10 June	Avaunt	170 g ha^{-1}	18 June	Mavrik	0.20 l ha^{-1}				0	x
P07	untr	24 May	Plenum	150 g ha^{-1}	31 May	Avaunt	0.20 l ha^{-1}	9 June	Plenum	150 g ha^{-1}	0	x
P07	treat	30 May	Avaunt	170 g ha^{-1}	8 June	Plenum	150 g ha^{-1}	17 June	Mavrik	0.25 l ha^{-1}	0	x

*untr, untreated; treat, clothianidin treated.
§swarm, swarmed; supers, superseded. Refers to the number of colonies per field that swarmed or superseded during the experiment.
‡Winter loss (between 05 September 2013 and April 2014) refers to number of colonies lost per field out of those deemed strong enough to overwinter by the assessing beekeeper.

Supplementary Table 9 | Observed changes in significance level and test statistics when excluding fields spray-treated with Biscaya from the analyses

**Model***	**Model type****	**Effect measure**	**Biscaya field**	**Estimate#**	**Degrees of freedom**	**$\chi 2$ or *F* value**	***P*§**
**Colony development**							
Number of adult bees	Full model	Seed-treatment x Bloom	Included	206	1, 110	*F*=4.22	**0.042**
			Excluded	148	1, 101	*F*=2.12	0.149
Honey production	Full model	Year	Included	-1.378	1, 18	*F*=4.47	**0.049**
			Excluded	-1.408	1, 16	*F*=3.68	0.073
**Pathogen prevalence**							
Nosema ceranae	Full model	Bloom x Year	Included	0.395	1	$\chi 2$=3.30	0.069
			Excluded	0.486	1	$\chi 2$=4.48	**0.034**
**Pathogen abundance**							
Aphid lethal paralysis virus	Full model	Seed-treatment x Bloom	Included	-0.567	1, 40	*F*=3.80	**0.039**
			Excluded	-0.434	1, 39	*F*=2.72	0.107
		Bloom x Year	Included	0.453	1, 40	*F*=3.49	0.096
			Excluded	0.983	1, 39	*F*=4.96	**0.032**
Chronic bee paralysis virus	2014	Seed-treatment x Bloom	Included	-0.691	1, 22	*F*=2.31	0.143
			Excluded	-0.983	1, 20	*F*=4.32	**0.050**

* Species names are spelled in italics.
** Full model: n = 14, clothianidin-treated; n =12, control fields; repeated measurements. 2014: n = 6, clothianidin-treated; n = 4 control fields.
# Main effects/interactions were estimated using sum-to-zero contrasts and the deviation of the second level (after, clothianidin) of each factor (Bloom: before/after, Seed-treatment: control/clothianidin) from to the grand mean (intercept) is presented.
§ P values < 0.05 are highlighted in bold.

Supplementary Table 10 | Virus primers. Details of the qPCR assays (RNA) for the pathogenic viruses screened in this study.

Pathogen/Parasite*	Abbreviation	Primers	Sequence '5-'3	Reference
Acute bee paralysis virus	ABPV	ABPV-F6548<br>KIABPV-B6707	TCATACCTGCCGATCAAG<br>CTGAATAATACTGTGCGTATC	[2]
Aphid lethal paralysis virus	ALPV	qALP-R6046<br>qALP-F5854	GCAGCACCGGAAACGTTTTTATGG<br>ACACCATAGTTCGCGAAGAACGCA	[2]
Black queen cell virus	BQCV	BQCV-qF7893<br>BQCV-qB8150	AGTGGCGGAGATGTATGC<br>GGAGGTGAAGTGGCTATATC	[2]
Big Sioux River virus	BSRV	qBSRV-R6134<br>qBSRV-F5853	CCCGCGATATAATTGCGTTTGTGAGC<br>GCGCCTATTTTCTGCAGCGCC	[3]
Chronic bee paralysis virus	CBPV	CBPV1-qF1818<br>CBPV1-qB2077	CAACCTGCCTCAACACAG<br>AATCTGGCAAGGTTGACTGG	[2]
Deformed wing virus type-A	DWV-A	DWV-F8668<br>DWV-B8757	TTCATTAAAGCCACCTGGAACATC<br>TTTCCTCATTAACTGTGTCGTTGA	[4]
Deformed wing virus type-B	DWV-B	VaDV-F1409<br>DWV-B1806	GCCCTGTTCAAGAACATG<br>CTTTTCTAATTCAACTTCACC	[2]
Israeli acute paralysis virus	IAPV	IAPV-F6627<br>KIABPV-B6707	CCATGCCTGGCGATTCAC<br>CTGAATAATACTGTGCGTATC	[2]
Kashmir bee virus	KBV	KBV-F6639<br>KIABPV-B6707	CCATACCTGCTGATAACC<br>CTGAATAATACTGTGCGTATC	[2]
Lake Sinai virus strains 1	LSV-1	qLSV1-R2743<br>qLSV1-F2569	GGGACGCAGCACGATGCTCA<br>AGAGGTTGCACGGCAGCATG	[3]
Lake Sinai virus strains 2	LSV-2	qLSV2-R1947<br>qLSV2-F1722	GCGGTGTCGATCTCGCGGAC<br>CGTGCTGAGGCCACGGTTGT	[3]
Slow bee paralysis virus	SBPV	SPV-F3177<br>SPV-B3363	GCGCTTTAGTTCAATTGCC<br>ATTATAGGACGTGAAAATATAC	[5]
Sac brood virus	SBV	SBV-qF3164<br>SBV-qB3461	TTGGAACTACGCATTCTCTG<br>GCTCTAACCTCGCATCAAC	[2]

* Species names are spelled in italics.

Supplementary Table 11 | Parasite and microbe primers. Details of the qPCR assays (DNA) for the pathogenic and non-pathogenic organisms screened in this study.

Pathogen/Parasite	Abbreviation	Primers	Sequence '5-'3	Reference
Gilliamella apicola	Gilliamella	Gilliam-16S-F	GTAACATGAGTGCTTGCACT	**This study**
		Gilliam-16S-R	CGCATGGCCCGAAGG	
Snodgrassella alvi	Snodgrassella	Snodgras-16S-F	ACGGAGAGCTTGCTCTC	**This study**
		Snodgras-16S-R	AAATAACGCGAGGTCTTTCGA	
Nosema apis	N. apis	forward	CTAGTATATTTGAATATTGTTTACAATGG	[6]
		reverse	GTCGCTATGATCGCTTGCC	
Nosema ceranae	N. ceranae	forward	TATTGTAGAGAGGTGGGAGATT	[6]
		reverse	GTCGCTATGATCGCTTGCC	

Supplementary Table 12 | Primers of the RT-qPCR assays for the immune defense and internal reference genes screened for the study

Immune defense gene	Abbreviation	DNA/RNA analysis	Primers	Sequence '5-'3	Reference
	Amel\LRR	RNA	forward	TAGTGAAATCTAGACCTC	This study
			reverse	ATGCAAAGAGCTATCATCA	
	Apidaecin	RNA	forward	TTTTGCCTTAGCAATTCTTGTTG	[7]
			reverse	GAAGGTCGAGTAGGCGGATCT	
	cSP33	RNA	forward	CGTCGGTGGTAAAGCGGCGA	[8]
			reverse	AACGGCGACCAACGTTGCCA	
	dorsal-1A	RNA	forward	TCGGATGGTGCTACGAGCGA	[8]
			reverse	AGCATGCTTCTCAGCTTCTGCCT	
	Eater-like	RNA	forward	GGCGAGTGCACCGGCTTGAA	[8]
			reverse	GCGCCATCGCGTCATAGCCA	
	NimC2	RNA	forward	GCGTGGAGGACGGGAAACCG	[8]
			reverse	ACATCGATGGCAGAGCGGCG	
	PGRP-S2	RNA	forward	GGCCACACACCAAATGCAGCAG	[8]
			reverse	CGAGGACCAGTGTGGCCATGT	
	SPH51	RNA	forward	TGGCAATTGTCTTTGCGGGCG	[8]
			reverse	TACTTCCGCCGCCGTTACGC	

Supplementary Table 13 | Observed changes in significance level and test statistics when including colonies that lost their queen or swarmed

Model*	Model type**	Effect measure	Swarmed/Queen loss	Estimate#	Degrees of freedom	*χ2* or *F* value	*P*§
**Colony development**							
Number of capped brood cells	Full model	Seed-treatment x Bloom x Year	Excluded	276	1, 110	*F*=6.26	**0.014**
			Included	206	1, 131	*F*=3.07	0.084
	2014	Seed-treatment x Bloom	Excluded	448	1, 35	*F*=5.02	**0.032**
			Included	410	1, 38	*F*=3.59	0.066
Number of adult bees	Full model	Seed-treatment x Bloom	Excluded	206	1, 110	*F*=4.22	**0.042**
			Included	130	1, 131	*F*=1.69	0.195
**Pathogen prevalence**							
Nosema ceranae	Full model	Bloom x Year	Excluded	0.395	1	*χ2*=3.30	0.069
			Included	0.513	1	*χ2*=6.11	**0.013**
**Pathogen abundance**							
Black queen cell virus	Full model	Seed-treatment	Excluded	-0.196	1, 18	*F*=5.25	**0.034**
			Included	-0.172	1, 20	*F*=3.66	0.070
Chronic bee paralysis virus	2014	Bloom	Excluded	-0.898	1, 22	*F*=3.91	0.061
			Included	-1.013	1, 25	*F*=5.79	**0.024**
Kashmir bee virus	2014	Bloom	Excluded	-0.777	1, 22	*F*=3.80	0.064
			Included	-0.838	1, 23	*F*=4.72	**0.040**
Snodgrassella alvi	Full model	Bloom x Year	Excluded	0.097	1, 110	*F*=4,69	**0.033**
			Included	0.086	1, 131	*F*=3.03	0.084
Varroa destructor	Full model	Seed-treatment x Bloom	Excluded	-0.069	1, 74	*F*=2.40	0.126
			Included	-0.101	1, 85	*F*=5.51	**0.021**

* Species names are spelled in italics

** Full model: n = 14, clothianidin-treated; n =12, control fields; repeated measurements. 2014: n = 6, clothianidin-treated; n = 4 control fields.

# Main effects/interactions were estimated using sum-to-zero contrasts and the deviation of the second level (after, clothianidin) of each factor (Bloom: before/after, Seed-treatment: control/clothianidin) from to the grand mean (intercept) is presented.

§ P values < 0.05 are highlighted in bold.

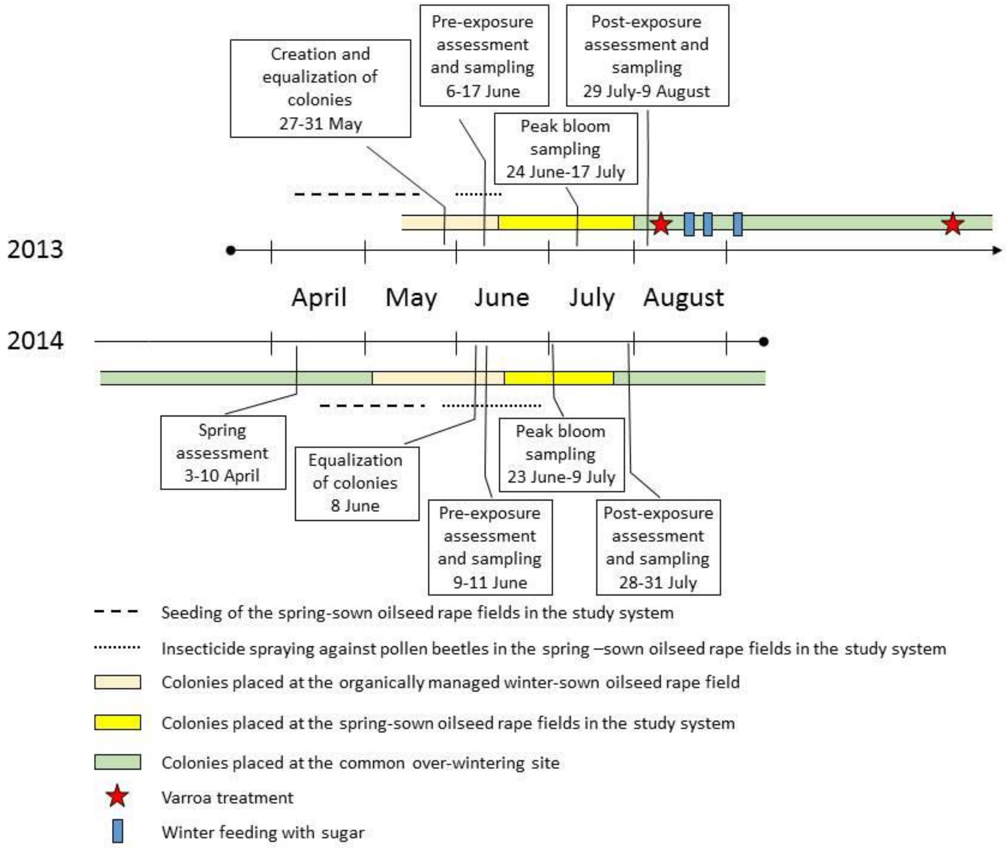

Supplementary Figure 1 | Experimental timeline. Overview of events within the study in both years (2013 and 2014), including honeybee colony creation and equalization, movement, treatment against *Varroa* mite and winter feeding of colonies with sugar, as well as seeding and insecticide spraying of the focal spring-sown oilseed rape fields. Information on dates of creation and equalization of the colonies and assessments are noted. Pre- and post-exposure assessments include measurements of colony strength (number of brood cells and adult bees per colony), hive weight to measure honey production and sampling of 100 bees for microbial assessments. Peak bloom sampling included collection of honeybees foraging in the oilseed rape fields, honeybees from the colonies and pollen from pollen traps on the hive entrance for clothianidin residue analysis and determination of pollen plant species origin.

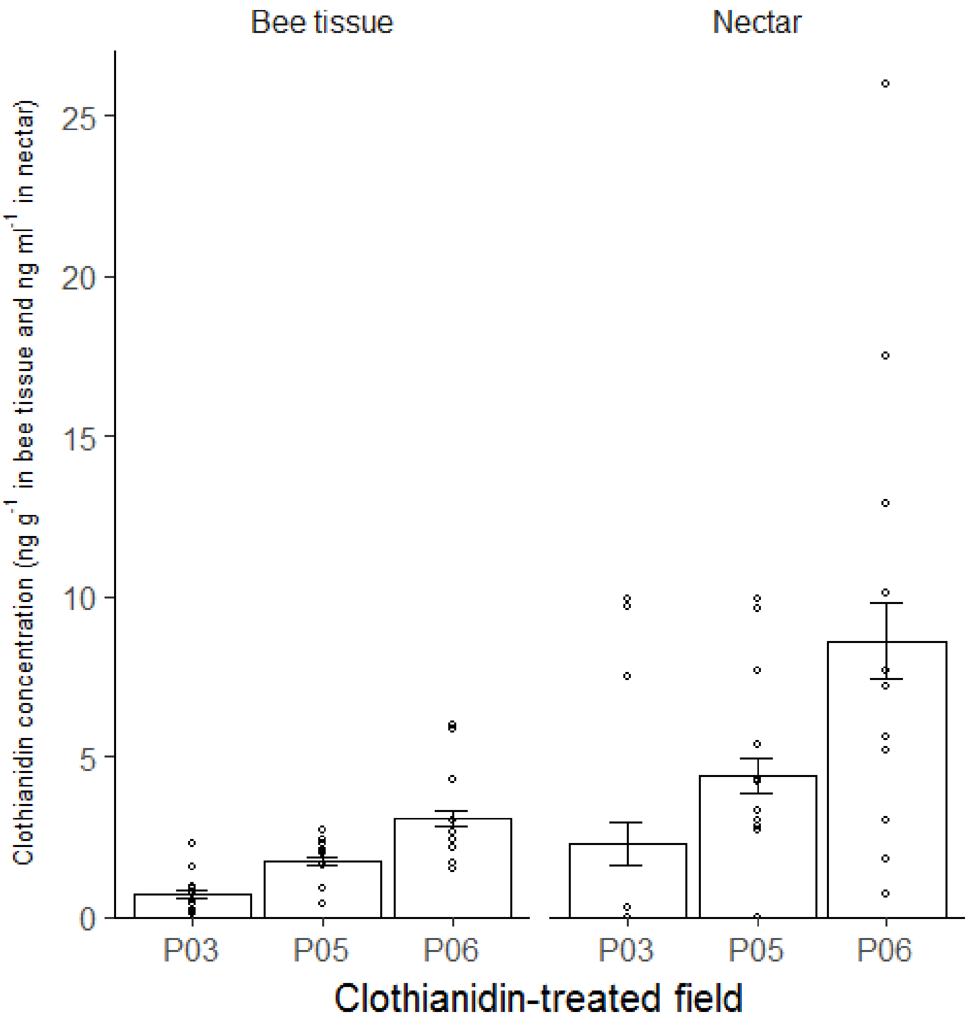

Supplementary Figure 2 | Clothianidin residues in individual bees. Mean clothianidin concentration (± 95 confidence limits) in honeybee tissue and nectar from their honey stomach in bees collected from hives adjacent to three 2013 oilseed rape fields (clothianidin-treated fields from P03, P05 and P05 pairs). Circles represent individual bees (N = 12 per field).

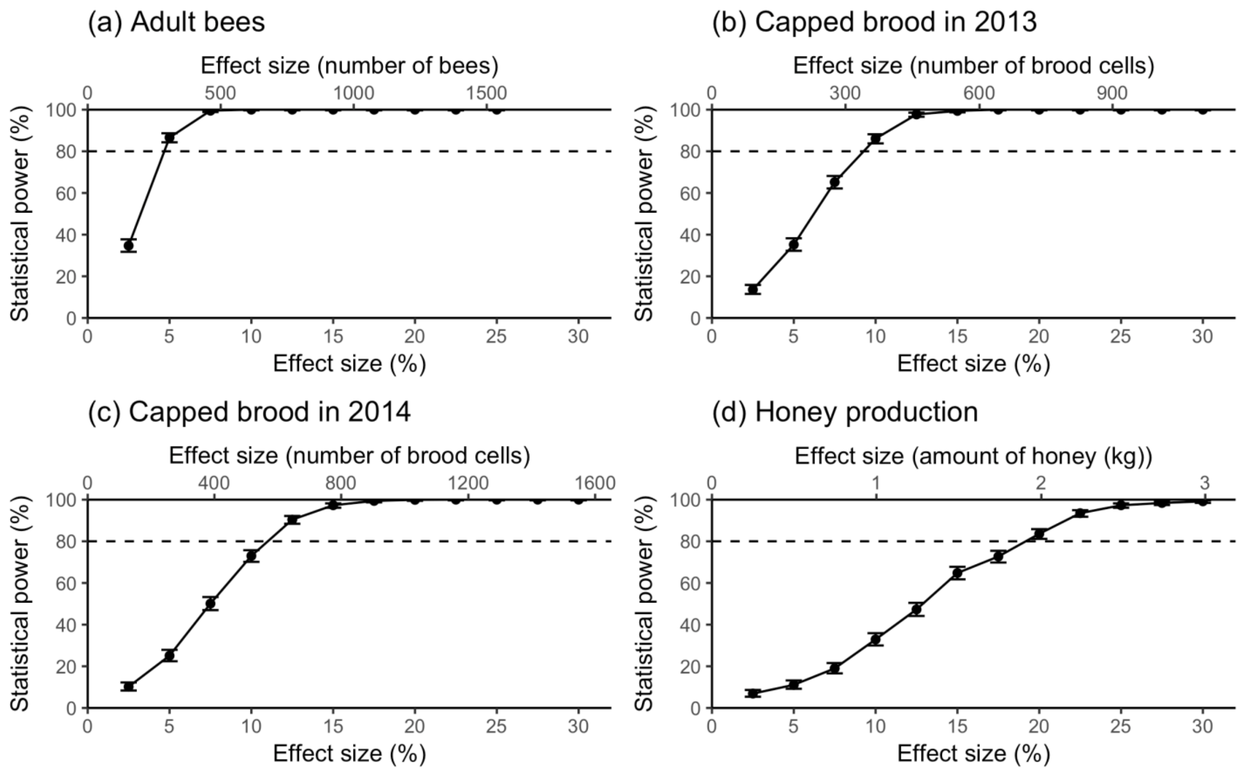

Supplementary Figure 3 | Power curves for honeybee colony strength and honey production. Relationship between statistical power and effect size estimated for interactive effects between clothianidin seed-treatment, bloom (before or after the oilseed rape bloom) and year (2013 and 2014) for the number of adult honeybees (full model) and the interactive effect between seed-treatment and bloom for the number of capped brood cell (separate models for the years). The effect size is expressed as the difference between treatments in the change in honeybee colony strength during the oilseed rape bloom in absolute terms and as a percentage of the mean number of adult honeybees or the number of capped brood cells in the control group before the oilseed rape bloom. For honey production, the relationship between statistical power and effect size estimated for amount of honey in kg in relation to seed-treatment is presented. The dashed horizontal line indicates a power of 80%.

Supplementary Methods

In order to determine neonicotinoid residues, pollen and nectar as well as honeybee tissue was analysed, pooled for each field (Supplementary Table 2). From each bee sample collected from the hive entrances during the peak bloom sampling (Supplementary Fig. 1), a subsample of 24 bees was weighed and homogenized with drying agent using a glass rod. From this homogenate a fraction corresponding to four bees was analysed. After addition of internal standard (IS) solution the homogenate was extracted twice using a 70:30 mixture of acetone and ethyl acetate (6 ml followed by 3 ml) under strong sonication (Vibra-Cell VCX 130, Sonics, USA). Extracts were further cleaned by dispersive solid phase extraction (SPE, using C18 and PSA) and evaporated to dryness at 40 °C under nitrogen gas flow. The extract residue was dissolved in 150 µl acetonitrile and analysed using liquid chromatography tandem mass spectrometry (LC-MS/MS) with positive electrospray ionization.

Pollen sample weights ranged between 0.025 and 0.104 g with an average of 0.057 g. Since the sample amounts were very small the entire sample was used for analysis. The extraction and clean-up steps were as for the pooled bee samples but in a downscaled format.

Honey stomachs were dissected in the field, pooled within each field and stored frozen pending analysis. Nectar samples were handled according to the Capillary Microsampling technique[9,10]. At the analytical laboratory nectar was transferred from the dissected honey stomachs, pooled, and then collected in glass capillaries with an exact volume of 16 µl. For three of the samples the nectar volume available was below 16 µl and an 8 µl capillary was used instead and a dilution factor applied. The capillaries were placed in 1 ml polypropylene tubes to which 32 µl IS

solution in acetonitrile, and an extra 32 µl acetonitrile, were added. The total dilution of the nectar sample was thus five times. After vigorously mixing of the tube followed by centrifugation, a volume of approximately 50-60 µl was transferred to an LC injection vial. Calibration samples at seven concentration levels (N = 2) and quality control samples at two levels (N = 3) were prepared in the same way by adding blank nectar (collected by bees and free from neonicotinoids) with a 16 µl capillary, 32 µl IS solution and finally a suitable amount of neonicotinoids via the extra 32 µl acetonitrile volume, i.e. a matrix match procedure.

Furthermore, to identify possible variation in neonicotinoid exposure of honeybees in different sites, we collected 12 honeybees per site from the entrance from three clothianidin-treated fields. Nectar was extracted from these honeybees and each bee and the nectar was analysed individually thereafter. Pesticide concentrations in bee tissue from single bees were determined after first removing the nectar from the honey stomach. The bee tissue was treated and extracted in the same way as for the pooled bee samples, but in a downscaled format and without the dispersive SPE clean-up step.

In the analytical laboratory, frozen bees were thawed and the abdomen containing the honey stomach was separated from the thorax with a scalpel. Nectar was collected after gently applying pressure with two fingers to the sides of the abdomen. Nectar coming out of the esophagus was collected in an 8 µl glass capillary and treated in the same way as described for the pooled nectar samples, but with an IS-volume of 40 µl, giving a total dilution of ten times. After filling the analytical capillary any surplus nectar from the honey stomach was gently pressed out and the remaining bee tissue was transferred to a sample tube for determination of neonicotinoid concentrations.

The LC-MS/MS (ESI+) method used for determination of the five neonicotinoids acetamiprid, imidacloprid, clothianidin, thiacloprid and thiamethoxam in extracts from bees, pollen and nectar samples was a modification of an accredited multi residue method for pesticides in water[11]. The main modifications was that 10 µl of acetonitrile extract was injected instead of 500 µl water, and that the stable isotope labelled internal standards clothianidin-D_3 (Teknolab AB, Kungsbacka, Sweden) and imidacloprid-D_4 (Dr Ehrenstorfer, Augsburg, Germany) were used. For the quantification of acetamiprid, thiacloprid and thiamethoxam D6-isoproturone was used as IS. Stock solutions of the investigated pesticides were certified by the manufacturers and dilutions from these stock solutions were made according to current standard operating procedures.

For the quantification of neonicotinoids in bee and pollen samples calibration curves in acetonitrile (seven concentration levels, duplicate injections; before and after samples) were used together with spiking experiments in blank bee and pollen matrix (two levels, N = 3). A factor for each compound and matrix, based on the measured and nominal concentrations of the spiked samples together with the sample weight, was used to calculate the concentrations in the study samples. The precision (RSD) in spiked bee samples was 2.4 – 19 % and in spiked pollen samples 0.9 – 18 %, for both concentration levels and all five neonicotinoids.

To confirm that the homogenates from the 24 bees were uniform, extra analyses were performed on subsamples (N = 4) from bee samples from two treated fields. The results showed a RSD of 4.6 and 8.5 %, respectively, for clothianidin.

For the nectar samples the concentrations were achieved directly from the calibration curve since study samples and calibration samples were both in nectar matrix and treated in exactly the same way. The quality control samples showed accuracies of 89-101 % and precisions (RSD) of 1.1 - 8.2 % for both concentration levels and all five neonicotinoids.

In all analytical runs matrix blanks and solvent blanks were analysed to confirm selectivity, and to check for carry-over between samples.

In a study separate from the field studies, the stability of neonicotinoids, as well as several other pesticides was investigated in frozen bees (-20 °C), living bees (for 1 h) and dead bees stored in room temperature (22 h), after individual feeding of bees with a sugar solution containing a pesticide mix[12]. All five neonicotinoids were stable (defined as degradation less than 20 %) in the freezer for up to 21 months and at room temperature for 22 hours. In living bees clothianidin was stable for 1 h while acetamiprid, imidacloprid, thiacloprid and thiamethoxam showed degradation with 44, 44, 43 and 22 %, respectively. Each estimate was based on triplicate samples and four bees per sample.

Supplementary References

1. SMHI. Reginal Climate Data. *Swedish Meteorological and Hydrological Institute* (2018). Available at: https://data.smhi.se/met/climate/time_series/month/vov_pdf/. (Accessed: 21st January 2018)

2. Locke, B., Forsgren, E., Fries, I. & de Miranda, J. R. Acaricide treatment affects viral dynamics in *Varroa destructor*-infested honey bee colonies via both host physiology and mite control. *Appl. Environ. Microbiol.* **78,** 227–35 (2012).

3. Runckel, C. *et al*. Temporal analysis of the honey bee microbiome reveals four novel viruses and easonal prevalence of known viruses, Nosema, and Crithidia. *PLoS One* **6**, e20656 (2011).

4. Forsgren, E., de Miranda, J. R., Isaksson, M., Wei, S. & Fries, I. Deformed wing virus associated with *Tropilaelaps mercedesae* infesting European honey bees (*Apis mellifera*). *Exp. Appl. Acarol.* **47,** 87–97 (2008).

5. de Miranda, J. R., Cordoni, G. & Budge, G. The *Acute bee paralysis virus-Kashmir bee virus-Israeli acute paralysis virus* complex. *J. Invertebr. Pathol.* **103,** S30–S47 (2010).

6. Forsgren, E. & Fries, I. Comparative virulence of *Nosema ceranae* and *Nosema apis* in individual European honey bees. *Vet. Parasitol.* **170,** 212–217 (2010).

7. Simone, M., Evans, J. D. & Spivak, M. Resin Collection and Social Immunity in Honey Bees. *Evolution (N. Y).* **63,** 3016–3022 (2009).

8. Nazzi, F. *et al.* Synergistic parasite-pathogen interactions mediated by host immunity can drive the collapse of honeybee colonies. *PLoS Pathog.* **8,** e1002735 (2012).

9. Jonsson, O. *et al.* Capillary microsampling of 25 µl blood for the determination of toxicokinetic parameters in regulatory studies in animals. *Bioanalysis* **4,** 661–674 (2012).

10. Jonsson, O. in Microsampling in Pharmaceutical Bioanalysis (doi:10.4155/EBO.13.399, *Future Science book series,* eds. Zane, P. & Emmons, G. T.) (2013).

11. Jansson, C. & Kreuger, J. Multiresidue analysis of 95 pesticides at low nanogram/liter levels in surface waters using online preconcentration and high performance liquid chromatography/tandem mass spectrometry. *J. AOAC Int.* **93,** 1732–47 (2010).

12. Jonsson, O. & Kreuger, J. Studie av växtskyddsmedels stabilitet i honungsbin samt av individvariation i fältexponering. CKB Report 2017:1 (in Swedish) Available at: https://www.slu.se/globalassets/ew/org/centrb/ckb/publikationer/ckb-rapporter/ckb-2017_1-stabilitet-och-individvariation-i-bin.pdf (2017).
