# Supplementary material for: Clothianidin seed-treatment has no detectable negative impact on honeybee colonies and their pathogens
Source: Nat Commun. 2019 Feb 11;10:692. doi: 10.1038/s41467-019-08523-4 (PMC6370849; doi:10.1038/s41467-019-08523-4)
Supplement: Supplementary file 3 — Description of Additional Supplementary Files [file 41467_2019_8523_MOESM3_ESM.pdf]

Description of Additional Supplementary Files

File Name: Supplementary Data 1
Description: Pathogen and parasite abundance in honeybee colonies in relation to clothianidin seed treatment, bloom (before and after oilseed rape bloom) and year (2013 or 2014).
