# Supplementary material for: Clothianidin seed-treatment has no detectable negative impact on honeybee colonies and their pathogens
Source: Nat Commun. 2019 Feb 11;10:692. doi: 10.1038/s41467-019-08523-4 (PMC6370849; doi:10.1038/s41467-019-08523-4)
Supplement: Supplementary file 5 — Reporting Summary [file 41467_2019_8523_MOESM5_ESM.pdf]

nature research

Corresponding author(s): Julia Osterman

Reporting Summary

Nature Research wishes to improve the reproducibility of the work that we publish. This form provides structure for consistency and transparency in reporting. For further information on Nature Research policies, see Authors & Referees and the Editorial Policy Checklist.

Statistical parameters

When statistical analyses are reported, confirm that the following items are present in the relevant location (e.g. figure legend, table legend, main text, or Methods section).

n/a	Confirmed	
☐	☒	The exact sample size (n) for each experimental group/condition, given as a discrete number and unit of measurement
☐	☒	An indication of whether measurements were taken from distinct samples or whether the same sample was measured repeatedly
☐	☒	The statistical test(s) used AND whether they are one- or two-sided<br>*Only common tests should be described solely by name; describe more complex techniques in the Methods section.*
☐	☒	A description of all covariates tested
☐	☒	A description of any assumptions or corrections, such as tests of normality and adjustment for multiple comparisons
☐	☒	A full description of the statistics including central tendency (e.g. means) or other basic estimates (e.g. regression coefficient) AND variation (e.g. standard deviation) or associated estimates of uncertainty (e.g. confidence intervals)
☐	☒	For null hypothesis testing, the test statistic (e.g. F, t, r) with confidence intervals, effect sizes, degrees of freedom and P value noted<br>*Give P values as exact values whenever suitable.*
☒	☐	For Bayesian analysis, information on the choice of priors and Markov chain Monte Carlo settings
☐	☒	For hierarchical and complex designs, identification of the appropriate level for tests and full reporting of outcomes
☒	☐	Estimates of effect sizes (e.g. Cohen's d, Pearson's r), indicating how they were calculated
☐	☒	Clearly defined error bars<br>*State explicitly what error bars represent (e.g. SD, SE, CI)*

Our web collection on statistics for biologists may be useful.

Software and code

Policy information about availability of computer code

Data collection	No software was used to collect data.
Data analysis	All data were analysed in R version 3.3.4 except for analyses adressing the verification of clothianidin exposure and land use, which were analysed using SAS 9.4 for Windows(SAS Institute Inc.).

For manuscripts utilizing custom algorithms or software that are central to the research but not yet described in published literature, software must be made available to editors/reviewers upon request. We strongly encourage code deposition in a community repository (e.g. GitHub). See the Nature Research guidelines for submitting code & software for further information.

Data

Policy information about availability of data

All manuscripts must include a data availability statement. This statement should provide the following information, where applicable:

- Accession codes, unique identifiers, or web links for publicly available datasets
- A list of figures that have associated raw data
- A description of any restrictions on data availability

The data supporting the findings of this study are available within the paper, its supplementary information files and/or Rundlöf et al. 15. The datasets generated during and/or analysed during the current study are available from the corresponding author on reasonable request.

Field-specific reporting

Please select the best fit for your research. If you are not sure, read the appropriate sections before making your selection.

☐ Life sciences ☐ Behavioural & social sciences ☒ Ecological, evolutionary & environmental sciences

For a reference copy of the document with all sections, see nature.com/authors/policies/ReportingSummary-flat.pdf

Ecological, evolutionary & environmental sciences study design

All studies must disclose on these points even when the disclosure is negative.

Study description	The study followed a randomized controlled design with paired fields of which one field per pair was randomly assigned to be sown with Elado-treated oilseed rape seeds, while the other field was sown with insecticide-free oilseed rape seeds. The experiment was conducted in 2013 and repeated a second year, but with reversed treatment allocation. In 2013, six honeybee colonies were placed per field (8 per treatment). After overwintering at a common location, the colonies were in 2014 randomly re-assigned to six clothianidin-treated and four control fields from the same farmers but with reverse treatment. In 2014, 4 colonies were placed per field.
Research sample	We investigated honeybee (Apis mellifera) colonies managed by a professional beekeeper.
Sampling strategy	The development of the honeybee colonies was measured in the field. Additonally 100 bees per colony were taken for microbial analysis both before and after the oilseed rape bloom period.
Data collection	Data on colony development measures were collected by a specialist trained beekeeper assistant, under supervision of Maj Rundlöf. The microbial data were collected by authors of this manuscript (Julia Osterman, Piero Onorati & Emilia Semberg). Land use data was extracted by Maj Rundlöf. Residues of neonicotinoids were analysed by Ove Jonsson.
Timing and spatial scale	Colonies were created and equalized between 27th and 31st of May. The first pre-exposure assessment and sampling was conducted between the 6th and the 17th of June. Peak bloom sampling was undertaken during 24th of June and the 17th of July and the post-exposure assessment during 29th of July and 9th of August in 2013.<br>In the second year of the experiment, a spring assessment was done between the 3rd and the 10th of April 2014. Furthermore, we conducted an equalization of the colonies (8th of June), a pre-exposure assessment (9-11 June), peak bloom sampling (23 June - 9 July) and a post-exposure assessment (28 - 31 July 2014). Further details are given in the manuscript.
Data exclusions	Colonies that swarmed during the oilseed rape bloom were excluded since swarming has a large effect on colony development and potentially also on pathogen levels.
Reproducibility	Both years of the experiment are reported in the manuscript. No additional attempts were conducted.
Randomization	In the first year of the experiment fields were paired according to geographical location and land use in their surroundings. Treatments were randomly assigned to fields within a pair. Colonies were randomly allocated to the fields. In the second year, some farms of the first year were re-used (although fields were different due to crop succession) but treatments were reversed. Colonies were, however, assigned to fields of the same treatment as in the first year with the exception of two control colonies from 2013 that were assigned to clothianidin-treated fields in 2014. Within treatments, colonies were randomly assigned to fields.
Blinding	Pseudonyms were used to mask treatment groups for people collecting data.

Did the study involve field work? ☒ Yes ☐ No

Field work, collection and transport

Field conditions	The colonies were placed in readily accessible locations on the edge of commercial oilseed rape farms in the southern part of Sweden, all with permission of the landowners. The experimental period was May 2013 to August 2014.
Location	The experiment was conducted in southern Sweden. The approximate locations of the oilseed rape fields are given in the manuscript.
Access and import/export	Access to the farms and fields was given expressly by the cooperating farmers and landowners. No personal data was collected. Only honeybees, pollen and nectar from the managed colonies was collected for analysis.
Disturbance	No major disturbance was caused by the placement of honeybee colonies in agricultural landscapes.

Reporting for specific materials, systems and methods

Materials & experimental systems

n/a	Involved in the study	
☒	☐	Unique biological materials
☒	☐	Antibodies
☒	☐	Eukaryotic cell lines
☒	☐	Palaeontology
☐	☒	Animals and other organisms
☒	☐	Human research participants

Methods

n/a	Involved in the study	
☒	☐	ChIP-seq
☒	☐	Flow cytometry
☒	☐	MRI-based neuroimaging

Animals and other organisms

Policy information about studies involving animals; ARRIVE guidelines recommended for reporting animal research

Laboratory animals	None
Wild animals	None
Field-collected samples	Worker honeybees (Apis mellifera) from managed colonies placed near commercial oilseed rape cultivations.
